# Bacterial diversity in ferruginous duricrust (*canga*) and the physicochemical variables affecting their prevalence, distribution and predicted metabolic pathways

**DOI:** 10.1007/s10482-026-02315-9

**Published:** 2026-04-24

**Authors:** Viviane Faria Morais Jotta, Carla Alessandra Silva, Glen Jasper Yupanqui García, Andrea Rodrigues Marques, Andria dos Santos Freitas, Aristóteles Góes-Neto, Fernanda Badotti

**Affiliations:** 1 Postgraduate Program in Product and Process Technology, Centro Federal de Educação Tecnológica de Minas Gerais (CEFET-MG), Belo Horizonte, Minas Gerais Brazil; 2 Postgraduate Program in Bioinformatics, Universidade Federal de Minas Gerais (UFMG), Belo Horizonte, Minas Gerais Brazil; 3 Department of Biological Sciences, Centro Federal de Educação Tecnológica de Minas Gerais (CEFET-MG), Belo Horizonte, Minas Gerais Brazil; 4 Department of Genetics, Universidade Federal of Minas Gerais, Belo Horizonte, Minas Gerais Brazil; 5 Department of Chemistry, Centro Federal de Educação Tecnológica de Minas Gerais (CEFET-MG), Nova Suiça, Av. Amazonas, 5.253, Belo Horizonte, Minas Gerais CEP: 30.421-169 Brazil

**Keywords:** Bacterial community, Environmental factors, Extremophiles, Ferruginous duricrust, Next-generation sequencing

## Abstract

The ferruginous duricrust (*canga*) in the Iron Quadrangle (IQ), Minas Gerais, Brazil, occurs within the *Campo Rupestre* and presents remarkable levels of endemism and species richness. Despite the recognized importance of microorganisms for the maintenance of this ecosystem, current molecular-based studies reveal that both the taxonomic composition and the ecological functions of the *canga* microbiome are undiscovered. In this study, eighteen samples of *canga* were collected from the Serra da Piedade State Natural Monument, and their taxonomic diversity was investigated using 16S rRNA metabarcoding. Additionally, the influence of physicochemical variables on microbial diversity and community structure was estimated using statistical tools. Most of the 856,667 reads clustered into ASVs corresponded to Bacteria (99.7%), and the most abundant of the 184 identified genera were *Conexibacter*, *Acidothermus* and *Bryobacter*. Microbial diversity was explained by a combination of physicochemical variables, such as organic matter (OM), iron (Fe), aluminum (Al) and pH, whereas microbial community structure was influenced by the concentrations of Fe, Al and the cation exchange capacity (CEC). Functional prediction analysis based on the main genera identified in the samples indicated that the denitrification pathway may play an important role in the ecosystem. The investigation of the genera and their metabolic pathways based on the literature revealed that they are unexplored and emphasized the biodiversity hotspot yet to be explored in ferruginous duricrust. Therefore, our results reinforce the importance of further studies in this environment, both for future biotechnological applications and for appropriate management and preservation actions.

**Supplementary Information:**

The online version contains supplementary material available at 10.1007/s10482-026-02315-9.

## Introduction

The *Campo Rupestre* is a megadiverse open grassland ecosystem that hosts 15% of all Brazilian vascular plants in 0.78% of the country’s territory. It is located at the interface of major biodiversity hotspots, the Atlantic Forest, the Cerrado, and the Caatinga domain (Bugado et al. 2025; Colli-Silva et al. 2019; Silveira et al. 2016). The *Campo Rupestre* of the Iron Quadrangle (IQ) occurs on both non-ferruginous and ferruginous substrates (locally known as *canga*) and is widely distributed at elevations ranging from approximately 900–2000 m (Mendonça et al. 2019). In Brazil, the term *canga* refers to ecosystems associated with a superficial iron crust (ferruginous duricrust) and is present in the states of Minas Gerais (Iron Quadrangle region) and Pará (Amazonia Carajas region) (Skirycz et al. 2014). Ferruginous duricrust is characterised by substrates with high concentrations of iron-rich oxides, and the vegetation in these areas has unique characteristics that significantly contribute to the regional and global biodiversity (Fernandes-Filho et al. 2022; Zappi et al. 2019).

Recent integrative studies combining microbial profiling with physicochemical characterization have highlighted the importance of evaluating both biological and chemical dimensions of environmental systems. Such studies have demonstrated that environmental parameters, such as elevated metal concentrations strongly shape microbial diversity patterns and adaptation strategies (Uluçay 2025, 2026). Finally, microorganisms isolated from extreme habitats have attracted increasing attention due to their potential for industrial enzyme production and biotechnological applications (Uluçay, Gormez, Ozic, 2022).

*Canga* areas are highly susceptible to degradation by mining activities, agricultural practices, erosion and the indiscriminate collection of species for commercial purposes. Climate change represents an additional concern, as it is estimated that half of the *Campo Rupestre* area will be lost by 2080 and that 84% of the species living on mountaintops are at serious risk of extinction (da Silva Arruda et al. 2024; Manes et al. 2021; Silveira et al. 2016). Despite the serious threat to its biodiversity, the microbiota that make up the *canga* ecosystem is overlooked using molecular biology tools.

DNA metabarcoding is a technique that uses next-generation sequencing (NGS) technology associated with specific biomarkers to identify multiple microbial taxa from complex environmental samples. 16S ribosomal RNA has been one of the most widely used gene targets in the study of bacterial biodiversity, as it allows the characterization of a community and its abundance (Orgiazzi et al. 2015; Vieira et al. 2018). While the diversity of Bacteria, Archaea, and Fungi in the soils of the Cerrado *stricto *sensu (forests and grasslands) and crop systems (pasture and soybean fields) has been evaluated by metabarcoding (Araujo et al. 2017), only a few studies have been performed in areas of *canga* (Skirycz et al. 2014; Vieira et al. 2018).

In this study, we investigated the taxonomic diversity of Bacteria and Archaea from samples collected from a preserved area of *canga* in the ferruginous *Campo Rupestre* (Serra da Piedade State Natural Monument) using 16S rRNA metabarcoding. Furthermore, 18 physicochemical variables and their influence on the microbial community structure were assessed using statistical modelling. Our aim was to contribute to the scarce knowledge about the microbial diversity inhabiting the *canga,* which is a promising environment for microbial exploration for the identification and discovery of new biological functions. Additionally, we conducted an extensive review of the literature on the physiological characteristics and metabolic capacities of the main bacterial genera identified. Notably, most of them belong to taxonomic groups poorly understood, with only a limited number of cultivated representatives, reinforcing that the *canga* biodiversity represents a valuable yet underexplored resource.

## Materials and methods

### Study area and soil sampling

**Fig. 1:**
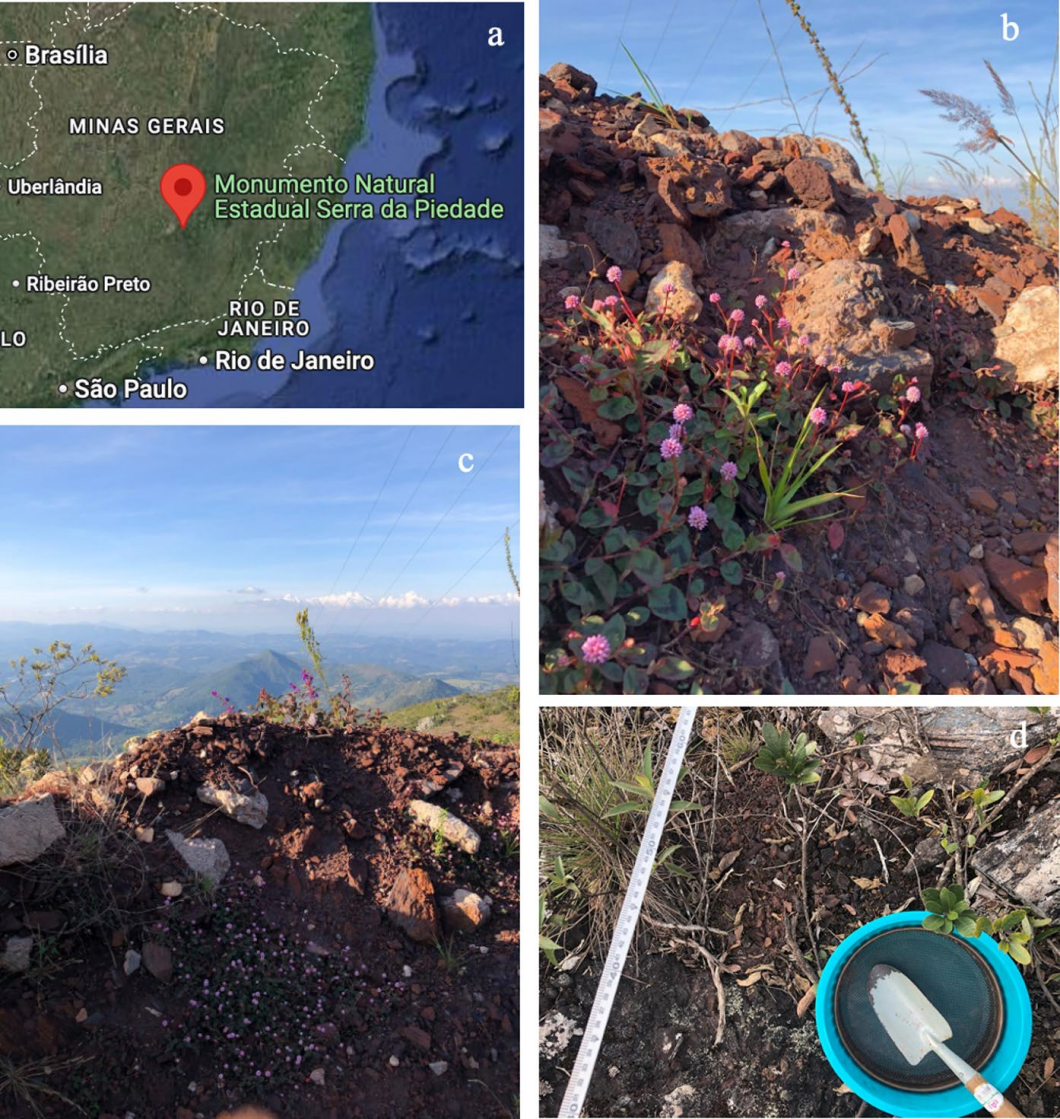
Geographical location of the Serra da Piedade State Natural Monument (MONAESP) in the Iron Quadrangle in Minas Gerais, Brazil (**a**). Landscape and natural vegetation in the *canga* area (**b** and **c**) and details of our sample collection (**d**)

Ferruginous duricrust samples were collected at the Serra da Piedade State Natural Monument (Monumento Natural Estadual da Serra da Piedade—MONAESP) in June 2021. The Serra da Piedade is situated at the northern border of the Iron Quadrangle, Minas Gerais, Brazil (19° 49′ 21,51″ S e 43° 40′ 38,22″ O) (Fig. 1a). This region represents an important site of geological heritage due to its iron formation of Paleoproterozoic age. Its scientific value as well as the beautiful landscapes have been acknowledged since the early nineteenth century and are currently included in the lists of natural and cultural heritage of the National Institute of Historic and Artistic Heritage (Instituto do Patrimônio Histórico e Artístico Nacional—IPHAN) (Fig. 1b, c and d).

**Fig. 2:**
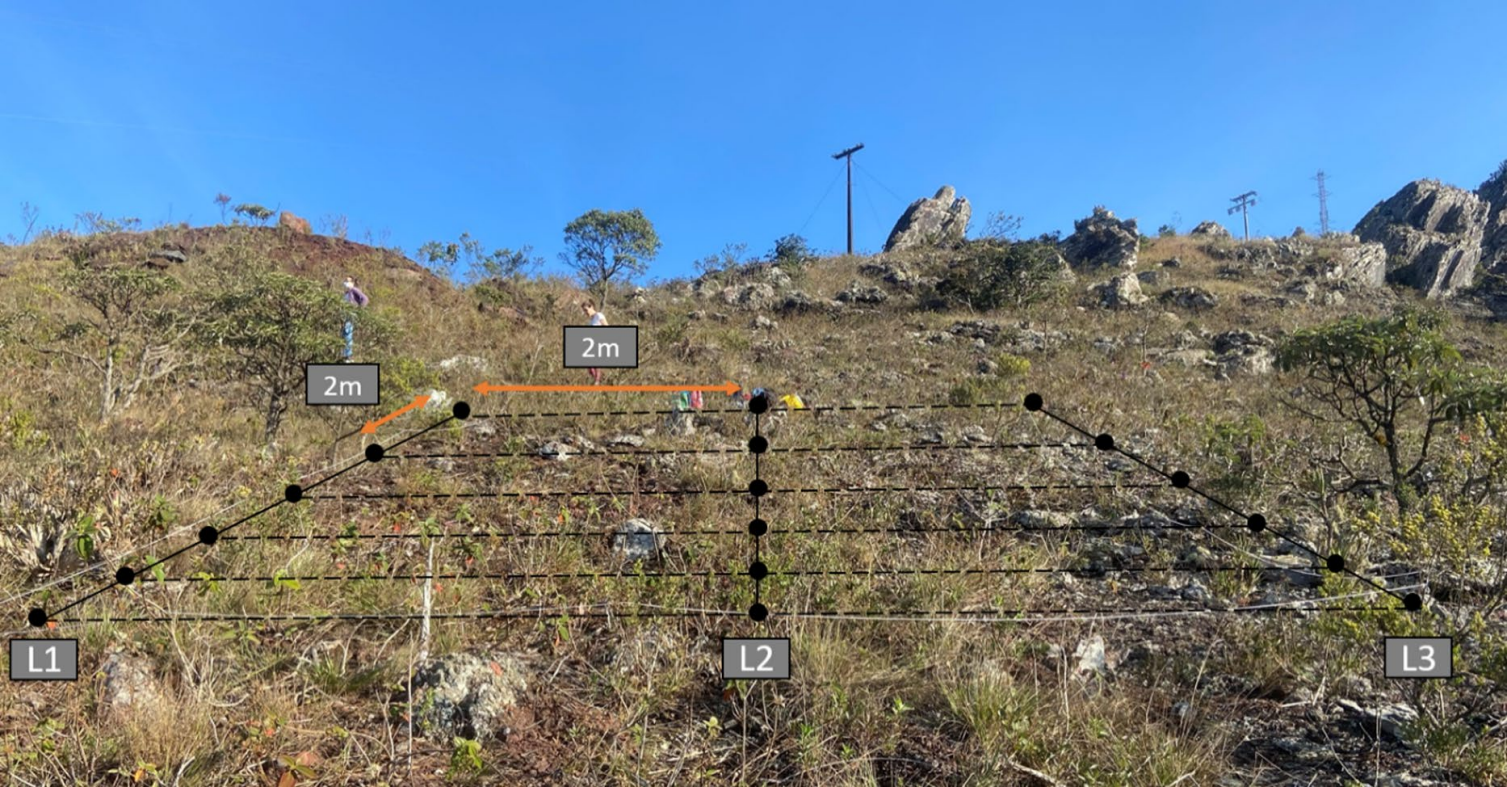
Sampling plan showing the three vertical transects (L1, L2 and L3) and the six samples collected from each transect, totalling the eighteen samples

The collection area was divided into three vertical transects (L1, L2, and L3), and six samples were collected at 2 m intervals, resulting in eighteen samples (Fig. 2). Intact samples were taken from 0 to 10 cm across the ferruginous duricrust ecosystem, which was the maximum sampling depth permitted by the substrate characteristics. The samples were sieved through a 2 mm mesh and placed into sterile plastic bags for physicochemical analysis, and into sterile Falcon tubes for further DNA extraction. The samples intended for molecular analyses were stored at − 20 °C, and those for physicochemical analyses were air-dried at 60 °C to a constant weight. Permission to access the park and collect samples was granted by the state government (n°004/2021) and can be accessed by the following link (http://sei.mg.gov.br/sei/controlador_externo.php?acao=documento_conferir&id_orgao_acesso_externo=0)—verification code 26,304,679 and CRC code 159C958D.

### Physicochemical analyses

Physicochemical analyses were performed at the Soil Analysis Laboratory of the Minas Gerais Institute of Agriculture (IMA). A 1 mol L^−1^ (M) KCl solution was used to extract the exchangeable cations (Al, Ca, and Mg), and the readings were performed by an atomic absorption spectrophotometer Varian aa220 serial number el98023275, SIPS diluter serial number el98023527 (Mulgrave, Victoria, Austrália). P, K and the microelements Cu, Fe, Mn and Zn were extracted using a solution containing 0.05 mol L^−1^ of hydrochloric acid and 0.0125 mol L^−1^ of sulfuric acid. The P content was estimated using the colorimetric method, the K using a flame photometer and the microelements by an atomic absorption spectrophotometer (Claessen et al. 1997). The cation exchange capacity (CEC) and the effective cation exchange capacity (CEC eff.) were estimated by the sum of the charges of the exchangeable equivalents of Na, K, Mg, Ca, Mn, Al, Fe, and H. Base sum (SB) was calculated by summing all basic exchangeable cations. Organic matter (OM) was estimated by titration of the excess dichromate in the solution with ammonium ferrous sulfate (0.033 mol L^−1^) using the colorimetric method (Walkley and Black 1934).

### DNA extraction and sequencing

The DNA was extracted from 250 mg of each sample using the DNeasy® PowerSoil® Pro Kit (Qiagen, Germany) following the manufacturer’s instructions. The DNA concentration and purity was measured using a NanoDrop spectrophotometer (NanoDrop Lite Plus, Thermo Fisher Scientific, Waltham, MA, USA).

PCR amplification and library preparation were performed by Zymo Research Corporation (Botucatu—SP, Brazil) using the Quick-16S™ NGS Library Prep Kit (Zymo Research, Irvine, CA, USA). The bacterial 16S primers, custom-designed by Zymo Research, target the V3–V4 region of the 16S rRNA gene to maximize coverage and sensitivity. The sequences of primers used were 341F (CCTACGGGRSGCAGCAG) and 806R (GGACTACHVGGGTWTCTAAT).

Final PCR products were quantified by qPCR fluorescence readings, pooled at equimolar concentrations, and purified using the Select-a-Size DNA Clean & Concentrator™ kit (Zymo Research). The pooled library was quantified with TapeStation® (Agilent Technologies, USA) and Qubit® fluorometer (Qubit 4, Thermo Fisher Scientific, Waltham, MA, USA). Sequencing was performed on an Illumina sequencing platform (MiSeq™, Illumina, San Diego, CA, USA) with a v3 reagent kit (600 cycles), generating 2 × 300 bp paired-end reads, and included a 10% PhiX spike-in as an internal control.

### Identification of core and satellite genera

The core and satellite species hypothesis, first introduced by Ilkka Hanski (1982), suggests that the species in a community can be classified into two distinct types, termed *core* and *satellite*. The first are locally abundant, widely distributed and often the dominant and the most successful in the community, whereas the satellites are rare and occur only at a few sites, and are often more susceptible to extinction. In this study, we classified the bacterial genera identified in all the samples as core, and those absent in at least one sample as satellite, regardless of their abundance. This analysis allowed us to visualize genera that were “hidden” by their low abundance.

### Bioinformatics

The quality of the raw sequencing reads was initially assessed via FastQC v0.11.9 (Andrews 2010). Adapter sequences were removed with Cutadapt v5.0 (Martin 2011). Forward and reverse reads were merged via VSEARCH v2.17.1 (Rognes et al. 2016) with the option –fastq_mergepairs. Quality filtering was performed with VSEARCH via the –fastq_filter command, retaining reads with a maximum expected error (–fastq_maxee) of 0.8 and a minimum length (–fastq_minlen) of 350 bp. Dereplication of sequences was carried out with VSEARCH via the –derep_fulllength option. Amplicon sequence variants (ASVs) were inferred via UNOISE3 (Edgar 2016) implemented in USEARCH v11, which performs error correction (*denoising*) and chimera removal as part of the ASV generation process. The ASV abundance table was generated with VSEARCH via usearch_global at 99% identity (id = 0.99). Taxonomic classification of ASVs was performed with the SINTAX algorithm (Edgar 2016) implemented in USEARCH via the SILVA reference database. The final abundance table integrating taxonomic classifications was generated with the custom script get_abundances_table_asv.py, available in the GitHub repository of the pipeline (https://github.com/LBMCF/pipeline-for-amplicon-analysis).

### Statistical analyses

#### Analyses of ecological data

All the statistical analyses were performed using software R (R Core Team 2025). The diversity of genera was estimated through taxonomic composition, abundance and diversity indices, which combine both richness and abundance. Rarefaction curves were built with the threshold set to the minimum library size, which in our dataset was 18,011 reads (AM6 sample).

The diversity was estimated using the Shannon index (H’), which was calculated as $\left( {H{\prime} = - \sum {ni/n \, ln\left( {ni/n} \right)} } \right)$, and the Simpson index $\left( {D = 1 - \sum {\left( {ni/n} \right)}^{2} } \right)$, where *ni* is the number of individuals of taxon *i*, and *n* is the total number of individuals in the sample (Colwell 2009). The homogeneity of genus abundance (Evenness) was estimated using Pielou’s formula (*J* = *H’/H’max*), where H’_max_ = ln(*S*) and *S* is the total number of genera (Legendre and Legendre 1998). The Shannon index was used to determine differences in diversity between the transects. The Shannon index values were normally distributed across all transects (L1: *p* = 0.269, L2: *p* = 0.903; L3: *p* = 0.373). Homogeneity of variance was confirmed via Levene’s test (*p* = 0.47). On the basis of these assumptions, one-way ANOVA was conducted, revealing a significant difference in diversity among transects (*p* = 0.02). A Tukey post hoc comparison test was performed to identify which transects differed significantly from each other (Zar 1996).

The data were standardized using the Hellinger transformation, and NMDS was subsequently performed to visualize patterns and groupings at the genus level on the basis of Bray‒Curtis dissimilarity. NMDS was run under a random starting configuration using the metaMDS function. To assess significant differences in community composition, PERMANOVA was conducted. The statistical significance of the differences between groups was tested using 999 permutations. This approach allowed us to evaluate whether the variation in genus composition between groups was greater than within groups, providing evidence for potential structural differences in the analysed community.

Community ecology analyses, including Hellinger transformation, NMDS, PERMANOVA, BETADISPER, diversity indices, and environmental fitting, were conducted using the Vegan package (Oksanen et al. 2025). Rarefaction and extrapolation analyses were performed using iNEXT (Hsieh et al. 2016). Data manipulation and visualization were carried out mainly with dplyr, tidyr, and ggplot2 (Wickham 2016), while univariate statistical tests were conducted using functions from stats, car, rstatix, and PMCMRplus.

#### Analyses of physicochemical data

Physicochemical variables were first tested for normality using the Shapiro–Wilk test. For variables that deviated from a normal distribution, non-parametric Kruskal–Wallis test (Hollander and Wolfe 1973), followed by Nemenyi post-hoc comparisons (Pohlert 2014) were applied. For variables that adhered to normality assumptions the one-way ANOVA with Tukey’s post-hoc test (Kassambara 2023) were used. No data transformations were performed on the physicochemical data, as the statistical approach was guided to the specific distribution of each variable.

#### Statistical modelling using taxonomical identification and physicochemical data

To model the relationships between *canga* physicochemical variables and microbial diversity, multiple linear regression was performed with Shannon diversity as the response variable. To assess multicollinearity among the physicochemical variables, Pearson’s correlation analysis was conducted. Correlation coefficients greater than 0.9 were considered indicative of strong multicollinearity between variables and were removed to avoid distortions in the regression estimates and ensure model stability (Online Resource 1).

A multiple linear regression model was initially constructed to predict Shannon diversity using the following environmental predictors: OM, Cu, Mn, Fe, Zn, pH, Al, Ca, Mg, and P. The model considering all these variables exhibited suboptimal performance, likely due to multicollinearity and overfitting. To improve model accuracy and parsimony, a stepwise backwards selection procedure was applied to identify the most informative variables. This process results in the exclusion of zinc (Zn) from the model, leading to improved interpretability and reduced variance in the estimated coefficients (Hastie and Pregibon 1992).

To model the relationships between physicochemical variables and microbial diversity, the community structure generated by the NMDS was associated with a matrix containing the environmental variables using the envfit() function. In addition, a constrained ordination approach was tested using Redundancy Analysis (RDA). Both the envfit() and RDA model were implemented using the vegan package in R. The scripts used in this study are publicly available at the following GitHub repository: https://github.com/alinebmv/Microbiome-soil.

### Functional prediction analyses

The functional prediction analyses were performed considering two distinct sets of amplicon sequence variants (ASVs): (i) the total set of ASVs detected in this study and (ii) a subset composed by the ASVs assigned to the four most abundant genera: *Conexibacter*, *Acidothermus, Bryobacter* and *Mycobacterium.* For this purpose, PICRUSt2 software version 2.6.2 (Douglas et al. 2020) was used. The ASVs abundance table and the FASTA file containing the representative sequences of the ASVs were used as inputs. Functional inference was based on the MetaCyc database (Caspi et al. 2020), resulting in a matrix containing the relative abundances of non-stratified metabolic pathways. This matrix was subsequently enriched with functional descriptions of each pathway, facilitating biological interpretation of the data. The generated functional profiles were then analysed using STAMP software version 2.1.3 (Parks et al. 2014) to identify differentially abundant pathways among the sampled transects. The Kruskal‒Wallis test was performed for comparisons between multiple groups, and the pair-by-pair differences were evaluated using the Tukey‒Kramer post hoc test with a 95% confidence level. The eta-squared (η2) value was used as a measure of effect magnitude. The results were filtered considering a *p*-value < 0.05 as the threshold for statistical significance. Since PICRUSt2 provides inferred functional profiles rather than directly measured functions no correction for multiple testing was applied; therefore, the results should be interpreted with caution. The results were visualized via hierarchical heatmaps with clustering by similarity.

## Results

### Sequencing analyses

A total of 1,707,694 merged reads were obtained from sequencing, with a mean value of 94,871 reads per sample. After quality filtering, 1,065,670 reads were obtained, with an average of 59,037 reads per sample. A total of 856,667 reads were clustered into ASVs, with 48,093 per sample. The mean percentage of reads used (the difference between the filtered reads and those clustered into ASVs) was 81.5% (Online Resource 2).

### Taxonomic identification

**Fig. 3:**
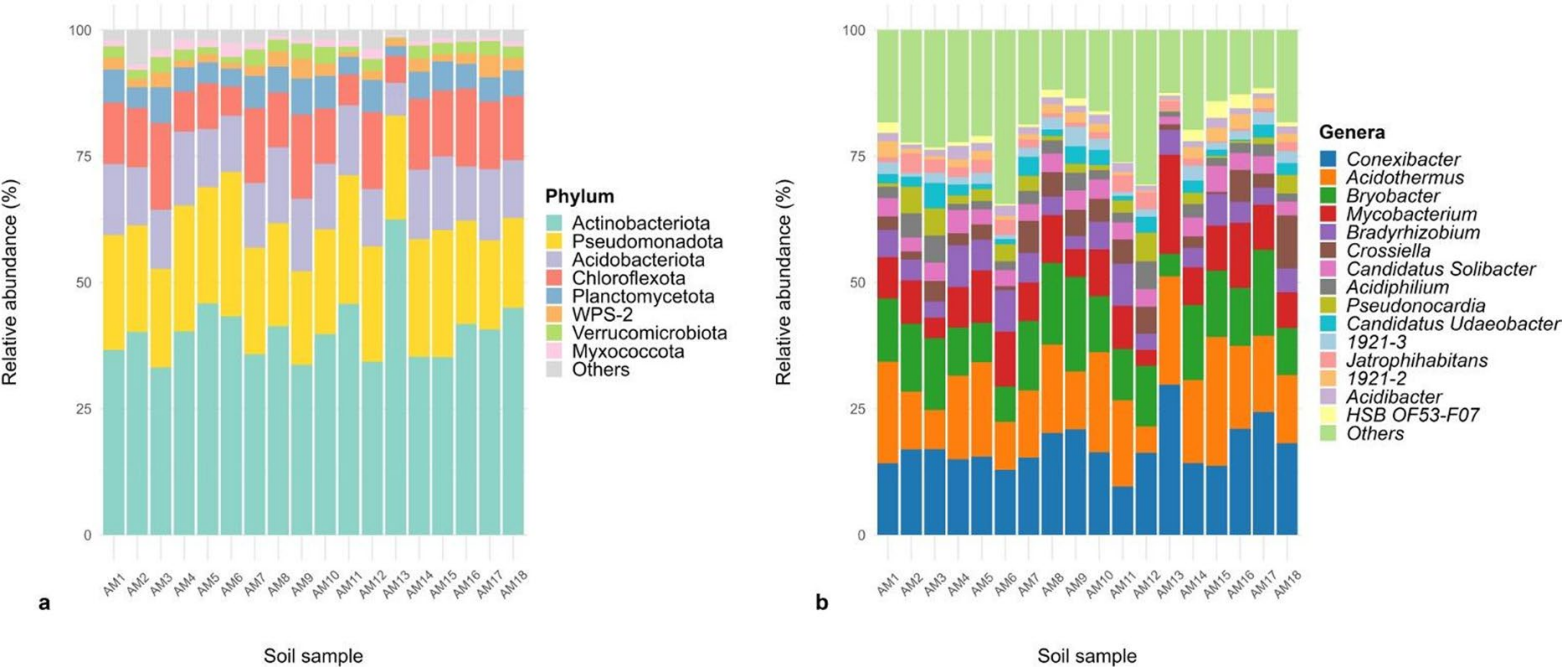
Relative abundance (%) of the main bacterial phyla (**a**) and genera (**b**) identified in the eighteen samples evaluated

Metataxonomic analysis revealed that most of the reads corresponded to Bacteria (99.7%), whereas only 0.3% corresponded to Archaea. Twenty-four phyla of Bacteria were identified, being Actinomycetota (40.3%), Pseudomonadota (21.8%), Acidobacteriota (12.6%) and Chloroflexota (12%) the most abundant (Fig. 3a). Two archaeal phyla were identified in the samples: Crenarchaeota (92.2%) and Thermoplasmatota (7.8%), and two families, Nitrososphaeraceae and Nitrosotaleaceae. Identification at the genus level for Archaea was not considered due to low accuracy.

A total of 184 genera of Bacteria were identified in the samples. *Conexibacter* (17.3%), *Acidothermus* (15.4%) and *Bryobacter* (12%) were the most abundant, followed by *Mycobacterium* (8.8%), *Bradyrhizobium* (5.1%) and *Crossiella* (3.8%) (Fig. 3b). The taxonomic composition and abundance of genera in the samples were similar, although some differences were noticed: AM13 had greater relative abundances of *Conexibacter* (29.7%), *Acidothermus* (21.5%) and *Mycobacterium* (19.6%) and lower abundance of *Bryobacter* (4.4%). In contrast, AM3, AM6, and AM12 had lower abundances of *Acidothermus* (7.8%, 9.5%, and 5.2%, respectively), whereas AM3 and AM12 had lower abundances of *Mycobacterium* (4.0% and 3.2%, respectively) (Fig. 3b). Three of the primary genera identified in the samples, *Acidothermus*, *Conexibacter* and *Mycobacterium*, are members of the phylum Actinomycetota, supporting the high abundance of this phylum in the samples, particularly in AM13. A complete list of the phyla and genera of Bacteria and Archaea identified and their relative abundances in the samples is available in Online Resource 3.

### Core and satellite communities

**Fig. 4:**
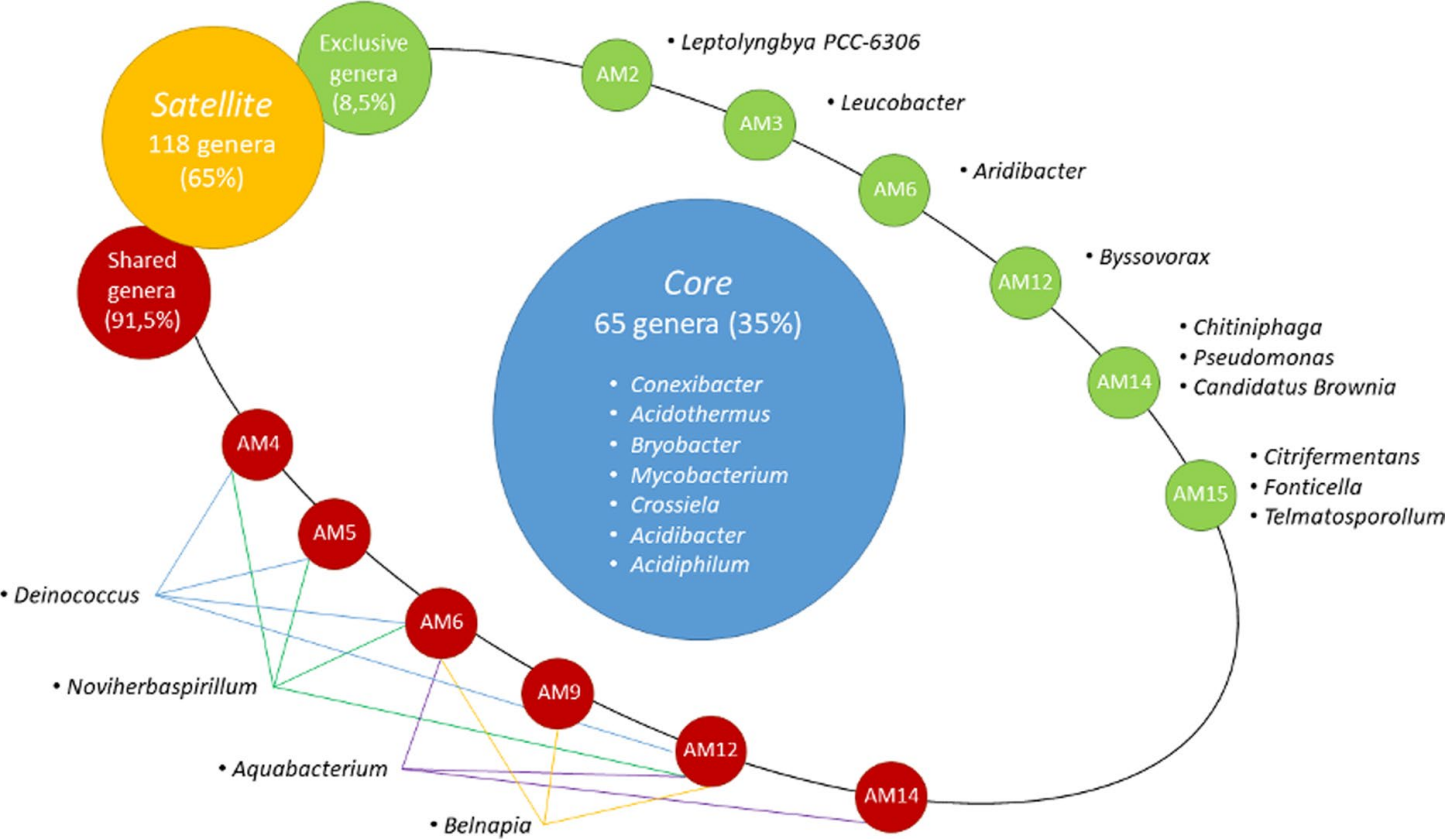
Representation of the main core and satellite genera of Bacteria and Archaea identified in the *canga* samples

The microbial common core among the samples was composed of 66 bacterial genera (35%) and included *Conexibacter*, *Acidothermus*, *Bryobacter*, *Mycobacterium*, *Crossiela*, *Acidibacter* and *Acidiphilum.* On the other hand, 118 (65%) genera were classified as satellite, such as *Deinococcus*, *Aquabacterium*, *Belnapia*, *Noviherbaspirillum, Arthrobacter* and *Rhodanobacter*. Among these, only 10 (8.5%) were exclusive to a single sample, such as *Chitiniphaga* and *Pseudomonas,* which were detected only in AM14, and *Citrifermentans*, *Fonticella* and *Telmatospirillum,* only found in AM15. Samples AM2, AM3, AM6 and AM12 presented one exclusive genus each: *Leptolyngbya*, *Leucobacter*, *Aridibacter* and *Byssovorax,* respectively (Fig. 4). Additionally, the Archaea family Nitrososphaeraceae was part of the core community, whereas Nitrosotaleaceae was part of the satellite community. The complete list of core and satellite communities is depicted in Online Resource 4.

### Diversity indices and statistical analyses

Rarefaction curves were used to evaluate whether the sequencing depth was sufficient to capture the low-abundance taxa. The curves for all the samples reached a plateau, indicating that the sequencing depth was sufficient (Online Resource 5).

**Fig. 5:**
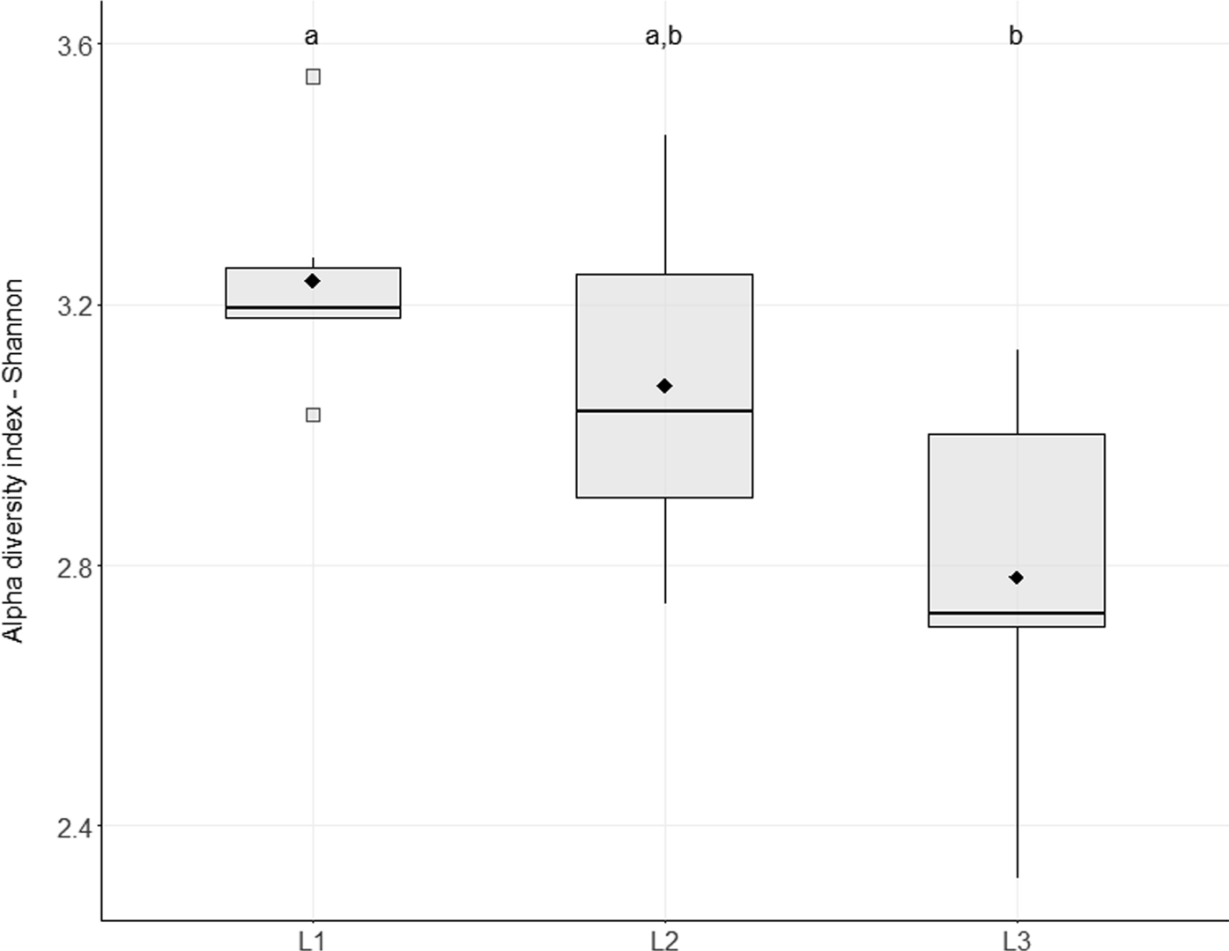
Boxplot based on the Shannon diversity index. The mean diversity value and the intrinsic variation for the samples from transects L1, L2 and L3 are shown. The outliers are represented as squares, and the mean values are represented as diamonds. Transects sharing the same letter do not differ significantly from each other, whereas transects with different letters are significantly different

The richness values ranged from 96 (AM13) to 158 (AM5). The diversity patterns, as measured by the Shannon index, varied from 2.32 (AM13) to 3.55 (AM6), whereas the Simpson index ranged from 0.82 in sample AM13 to 0.94 in AM6 (Online Resource 6). The L1 transect presented the highest overall diversity and the lowest within-transect variation, whereas the samples from L3 presented the lowest diversity values, and the samples from transects L2 and L3 presented greater intrinsic variation (Fig. 5). These patterns were supported by the statistical analysis of ANOVA followed by Tukey’s post hoc test, which revealed a significant difference in diversity between transects L1 and L3 (*p* = 0.02), whereas no significant differences were detected between L1 and L2 (*p* = 0.52) or L2 and L3 (*p* = 0.14). Additionally, evenness was consistently low across the samples, with values ranging from 0.07 to 0.12, indicating that the community structure was dominated by a few highly abundant genera, while the majority were present at low frequencies, reinforcing the *core* and *satellite* distributions mentioned above.

### Nonmetric multidimensional scaling (NMDS)

**Fig. 6:**
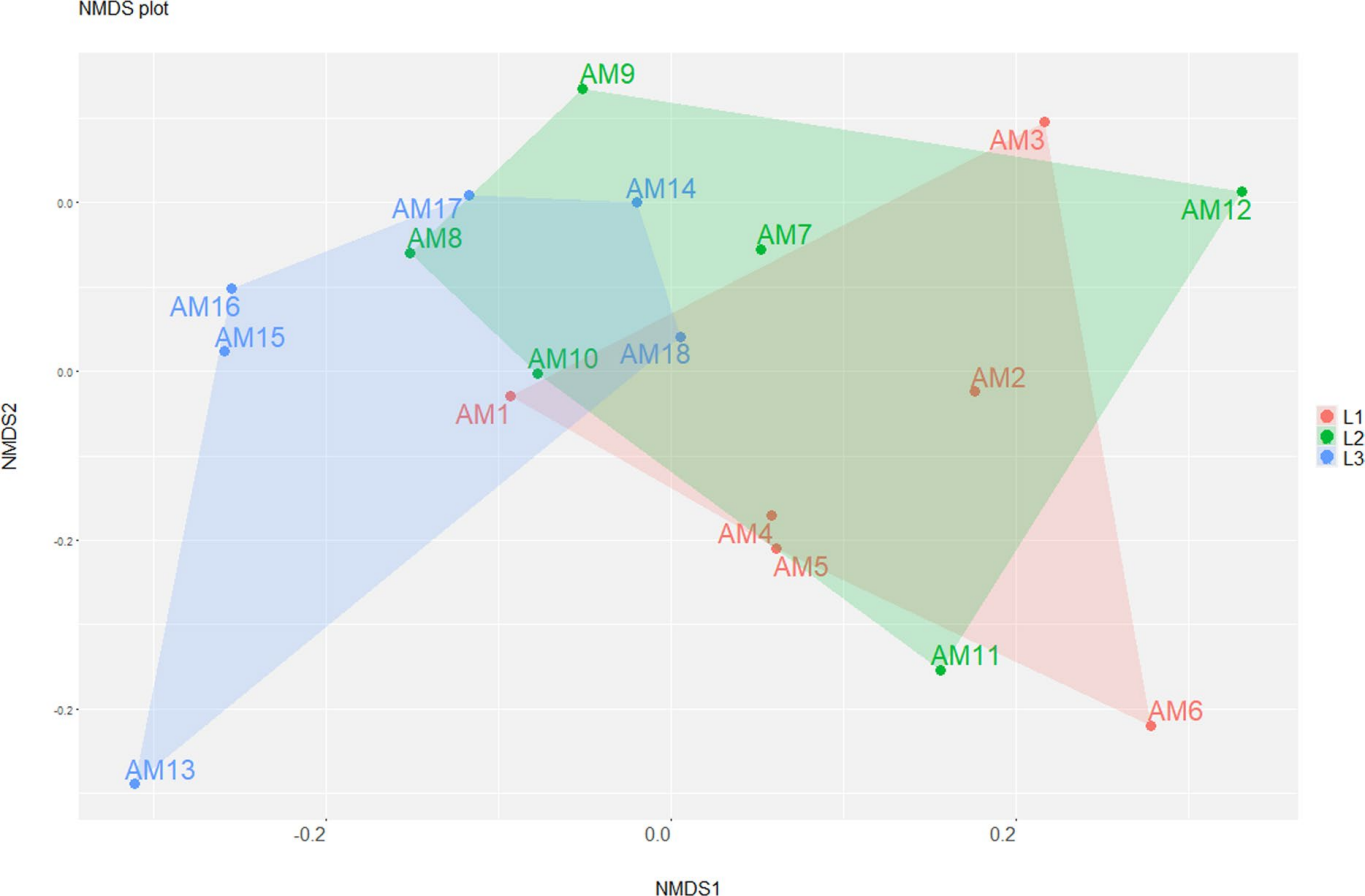
Nonmetric multidimensional scaling (NMDS) plot based on Bray‒Curtis dissimilarities derived from read abundances of bacterial genera recorded from the *canga* samples. The samples from transect L1 are represented in red, L2 in green, and L3 in blue

NMDS analysis showed a clear separation between genus-level communities across the different transects (stress = 0.063) (Fig. 6). ANOSIM revealed a weak and non-significant separation between groups (R = 0.11, *p* = 0.081), whereas PERMANOVA indicated that transects explained a significant portion of the variation in community composition (R^2^ = 0.23, *p* = 0.033). The discrepancy between ANOSIM and PERMANOVA is likely due to the different mathematical foundations of each test; while PERMANOVA partitions variance based on actual distances to detect shifts in community centroids, ANOSIM relies on ranks. This makes PERMANOVA more sensitive to subtle changes in composition, whereas ANOSIM is more conservative and focuses strictly on group dissimilarity. To confirm the robustness of the findings, a test for homogeneity of multivariate dispersions (BETADISPER) was conducted. The lack of significance in the dispersion analysis (F = 0.0635, *p* = 0.932) confirmed that the differences detected by PERMANOVA are due to shifts in community composition rather than artifacts of group variance.

### Physicochemical analyses

In addition to the taxonomic identification, physicochemical analyses were performed on the samples. The pH varied from 4.1 (AM4) to 5.5 (AM6 and AM11). The highest concentration of organic matter (OM) was detected in samples from transect L3 (5.86), followed by L2 (3.25) and L1 (2.67). Notably, sample AM13 presented the highest OM value (10.0 dag/kg), whereas AM3, AM6 (1.58 dag/kg) and AM9 (1.27 dag/kg) the lowest. Samples from transect L2 presented the highest sum of bases (SB), while the cation exchange capacity (CTC) and the effective cation exchange capacity (CTC-ef) were greater for transect L3. The aluminum saturation (m) varied from 3.02 (AM12) to 39.96 (AM15).

Samples from transect L3 presented the highest contents of C, N, P, K, Fe and Al. On the other hand, samples from transect L1 presented the poorest mineral content, whereas those from transect L2 presented high levels of Mn, Zn, Ca and Mg. Notably, the highest concentrations of all minerals mentioned for transect L3 were found in sample AM13, and for transect L2, in AM12 (Table 1).

**Table 1:** Results obtained for the physicochemical variables analysed in the eighteen *canga* samples studied

Sample	Transect	pH	OM	SB	CTC	CTC-ef	m	V	C	N
		(in H_2_O)	(dag/kg)	(cmolc/dm^3^)			%		(dag/kg)	
AM1	L1	4.2	2.48	0.95	4.61	1.15	17.55	20.61	1.44	0.12
AM2		4.6	5.31	1.14	7.66	1.71	33.12	14.91	3.08	0.24
AM3		5.1	1.58	0.74	3.14	0.85	13	23.68	0.92	0.09
AM4		4.1	2.36	0.86	4.24	1.07	19.78	20.27	1.37	0.12
AM5		5	2.72	1.01	4.71	1.18	14.55	21.43	1.58	0.14
AM6		5.5	1.58	0.93	3.03	1.01	7.99	30.73	0.92	0.09
*Average*		***4.75***	***2.67***	***1.05***	***4.57***	***1.16***	***17.67***	***20.18***	***1.55***	***0.13***
*SD*		***0.55***	***1.38***	***0.14***	***1.68***	***0.29***	***8.58***	***5.19***	***0.80***	***0.06***
AM7	L2	4.6	2.96	0.98	5.16	1.21	19.16	19	1.72	0.15
AM8		5	2.25	1.47	4.67	1.58	7.03	31.45	1.3	0.11
AM9		5	1.27	0.62	2.7	0.69	8.85	23.13	0.73	0.07
AM10		5.2	2.14	1.73	4.26	1.83	5.52	40.54	1.24	0.11
AM11		5.5	3.47	1.88	6.45	2.06	8.82	29.16	2.01	0.17
AM12		5	7.38	3.57	7.39	3.68	3.02	48.28	4.28	0.33
*Average*		***5.05***	***3.25***	***1.71***	***5.11***	***1.47***	***8.73***	***31.93***	***1.88***	***0.16***
*SD*		***0.29***	***2.16***	***1.03***	***1.66***	***1.02***	***5.56***	***10.90***	***1.25***	***0.09***
AM13	L3	5.4	10	1.78	16.45	2.83	37.1	10.83	5.8	0.45
AM14		4.8	5.16	1.16	7.12	1.62	28.66	16.24	2.99	0.23
AM15		4.5	6.83	1.27	10.57	2.12	39.96	12.05	3.96	0.31
AM16		4.5	6.3	1.12	7.79	1.71	34.27	14.43	3.65	0.28
AM17		4.7	4.28	1.02	7.18	1.57	35.35	14.15	2.48	0.21
AM18		4.6	2.6	0.73	4.15	0.91	20.04	17.5	1.51	0.13
*Average*		***4.75***	***5.86***	***1.18***	***8.88***	***1.79***	***32.56***	***14.2***	***3.4***	***0.27***
*SD*		***0.34***	***2.53***	***0.35***	***4.24***	***0.64***	***7.18***	***2.49***	***1.46***	***0.11***

Sample	Transect	P	K	Cu	Mn	Fe	Zn	Ca^2+^	Mg^2+^	Al^3+^
		(mg/dm^3^)						(cmolc/dm^3^)		
AM1	L1	2.3	17	3.1	7.1	141.7	2	0.78	0.12	0.2
AM2		5.3	40	2.3	3.8	219.2	2.2	0.88	0.16	0.57
AM3		2.3	12	0.3	3	117.9	1.7	0.63	0.08	0.11
AM4		2.9	15	2.5	3.5	138.1	1.9	0.71	0.12	0.21
AM5		4.6	37	4.4	12.6	133.2	3.1	0.76	0.16	0.17
AM6		3.7	13	0.3	4.1	110	2	0.78	0.12	0.08
*Average*		***3.52***	***22.33***	***2.15***	***5.68***	***143.35***	***2.15***	***0.76***	***0.13***	***0.22***
*SD*		***1.24***	***12.68***	***1.61***	***3.68***	***39.11***	***0.49***	***0.08***	***0.03***	***0.18***
AM7	L2	4.1	26	0.3	4.5	141.2	1.7	0.76	0.16	0.23
AM8		3.1	23	1.8	13.9	135.8	2.6	1.26	0.15	0.11
AM9		2.3	8	0.2	1.9	118.5	0.8	0.53	0.07	0.06
AM10		3.1	26	0.6	7.6	126.7	4.8	1.51	0.15	0.1
AM11		3.7	33	0.3	9	159.7	3.4	1.61	0.18	0.18
AM12		4.6	47	1.3	22.5	217.1	7.7	3.07	0.37	0.11
*Average*		***3.48***	***27.17***	***0.75***	***9.9***	***149.83***	***3.5***	***1.46***	***0.18***	***0.13***
*SD*		***0.82***	***12.77***	***0.65***	***7.40***	***35.81***	***2.48***	***0.90***	***0.10***	***0.06***
AM13	L3	4.8	60	0.4	7.5	769.9	3.1	1.44	0.19	1.05
AM14		5.3	39	1.6	6.4	233.2	2.4	0.91	0.15	0.46
AM15		3.7	46	1.7	5	623.1	2.5	0.96	0.2	0.85
AM16		3.7	36	0.3	12.1	278.6	2.3	0.88	0.15	0.59
AM17		4.1	30	0.3	3.7	223.9	1.8	0.81	0.13	0.56
AM18		2.5	18	2.4	4.3	115.9	1.4	0.6	0.07	0.18
*Average*		***4.02***	***38.17***	***1.12***	***6.5***	***374.1***	***2.25***	***0.93***	***0.15***	***0.62***
*SD*		***0.98***	***14.26***	***0.90***	***3.08***	***259.56***	***0.59***	***0.28***	***0.05***	***0.30***

The average and standard deviation (SD) values are shown in bold and italics.

### Statistical analyses of the physicochemical data

Since several variables deviated from a normal distribution, such as Al, C, Fe, K, OM, Mn, N and pH, non-parametric Kruskal–Wallis tests followed by Nemenyi post-hoc comparisons were performed, which indicated that only Al concentrations had statistical differences. For variables that adhered to normality assumptions, one-way ANOVA with Tukey’s post-hoc test was performed and revealed significant differences in the concentrations of CTC, m and V between the transects (Online Resource 7).

### Statistical modelling using taxonomic identification and physicochemical data

To estimate the variables influencing Shannon diversity, several models were tested and adjusted by comparing the predicted and actual values of the Shannon index. The final multiple linear regression included the following predictors:

$$ \begin{gathered}   {\mathrm{Shannon}}\, = \,0.{\mathrm{79}}\, + \,\left( {0.{\text{21 }}*{\text{ OM}}} \right)\, + \,\left( {0.{\text{11 }}*{\text{ Cu}}} \right)\, - \,\left( {0.0{\text{7 }}*{\mathrm{Mn}}} \right) \hfill \\   \quad \quad \quad \,\, - \,\left( {0.00{\text{3 }}*{\mathrm{Fe}}} \right)\, + \,\left( {0.{\text{53 }}*{\mathrm{pH}}} \right)\, - \,\left( {0.{\text{79 }}*{\mathrm{Al}}} \right)\, - \,\left( {0.{\text{63 }}*{\mathrm{Ca}}} \right)\, + \,\left( {{\mathrm{8}}.{\text{75 }}*{\mathrm{Mg}}} \right)\, - \,\left( {0.{\text{18 }}*{\mathrm{P}}} \right) \hfill \\  \end{gathered}  $$

This model demonstrated a Spearman correlation of *r* = 0.85 between the observed and predicted Shannon values. Additionally, the model had an RMSE of 0.18, indicating a relatively low average deviation between the predicted and actual values. However, given the low sample size and the high number of environmental predictors evaluated, these results should be interpreted cautiously, and the model should be regarded as exploratory rather than broadly predictive.

**Fig. 7:**
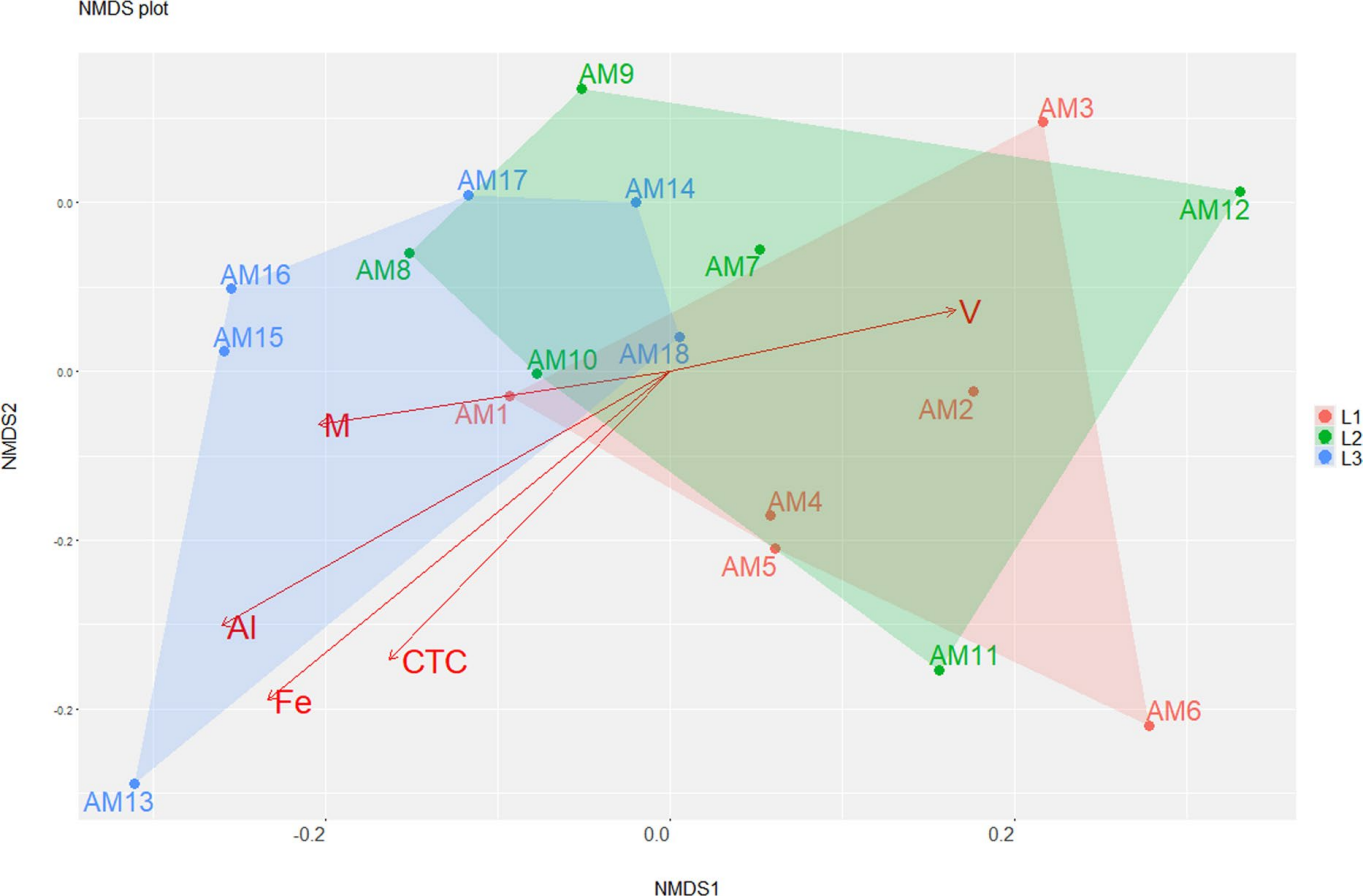
Nonmetric multidimensional scaling (NMDS) of the microbial community associated with the physicochemical data using envfit(). *Canga* samples are located according to the transects

Finally, taxonomic identification and physicochemical data were combined to evaluate the influence of physicochemical variables on community structure. For this purpose, two approaches were used, unconstrained, using envfit() and constrained, using RDA. The results were similar for both and the variables significant for the model were Fe, Al, CTC, m and V, and most of them influenced the microbial community of samples from transect L3 (Fig. 7).

### Functional prediction

**Fig. 8:**
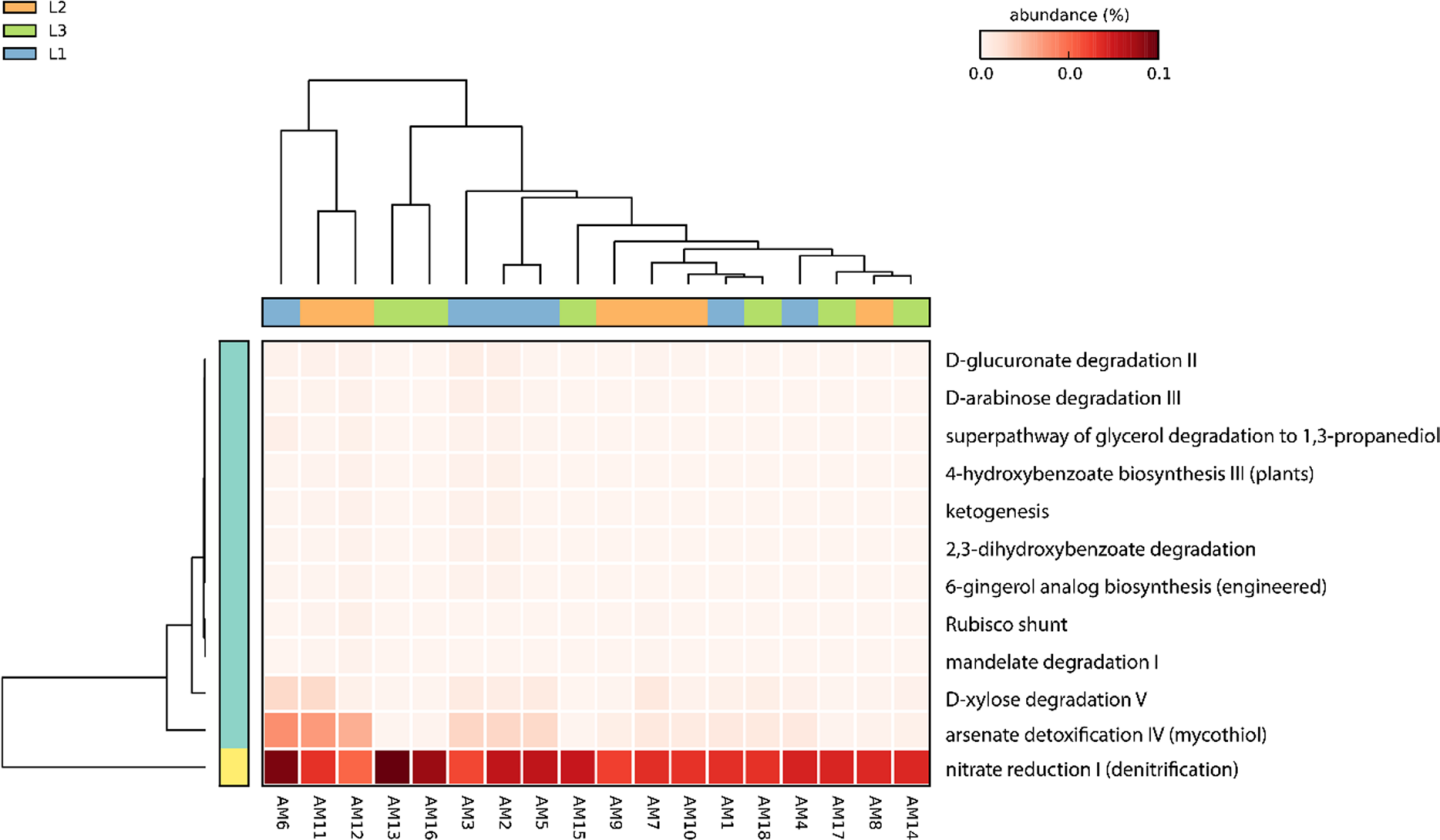
Heatmap of functional prediction based on the four dominant genera identified in the samples. The prediction included the phylogenetic positioning of the sequences, the reconstruction of ancestral states and the prediction of the relative abundance of genes and metabolic pathways per sample

The functional prediction analysis based on the complete dataset of ASVs resulted in 39 metabolic pathways significantly different between the transects, being the GDP-mannose biosynthesis and the pentose phosphate pathway the most abundant (Online Resource 8). When the subset of ASVs assigned to the four most abundant genera (*Conexibacter*, *Acidothermus, Bryobacter* and *Mycobacterium)* was evaluated, 12 pathways were identified, being the denitrification pathway the most abundant (Fig. 8).

## Discussion

### Bacterial diversity and ecological functions

The three main phyla identified in our samples, Actinomycetota, Pseudomonadota and Acidobacteria, are commonly found in soil microbial diversity studies (Araujo et al. 2017; Bandeira et al. 2024; Vieira et al. 2018).

Actinomycetota (previously Actinobacteria) represented more than 40% of the sequences recovered from our samples, and the main representative genera within this phylum were *Conexibacter*, *Acidothermus*, *Mycobacterium* and *Crossiela*. Actinomycetota play important roles in nutrient recycling, soil organic matter formation and dissimilatory reduction of ferric iron (Devi et al. 2022; Jones and Johnson 2015). Bacterial communities in arid ecosystems are frequently dominated by members of this phylum (Neilson et al. 2012), which explains its high abundance in *canga*, an ecosystem exposed to prolonged droughts.

Pseudomonadota (previously Proteobacteria) is one of the largest and most versatile phyla known, encompassing a wide variety of morphological, physiological and metabolic characteristics. They are important maintainers of the carbon, nitrogen and sulfur cycles. Owing to their versatility, members of Pseudomonadota can be found in almost any environment worldwide, including extreme ones (Shu et al. 2022). This was the second most abundant phylum in our samples (21.8%), and was mainly represented by the genera *Bradyrhizobium*, *Acidiphilium*, *Acidibacter* and *Roseiarcus*. Despite being a well-characterised phylum with many cultured representatives, our knowledge remains far from adequate. A prominent study conducted by Spain et al. (2009) analysed 13,001 nearly full-length 16S rRNA gene clones and identified 15 novel order-level and 48 novel family-level Pseudomonadotal lineages, most belonging to orders and families with no cultivated representatives.

Acidobacteria are globally distributed and yet one of the least understood phylogenetic groups in the domain Bacteria (Janssen 2006; Sikorski et al. 2022). Members of this phylum have been abundantly detected in soils and sediments, comprising 10 to 50% of the total bacterial 16S rRNA gene sequences (Barns et al. 2007; Lee et al. 2008), corroborating to the 12.5% of sequences identified in our samples. Despite the success in detecting Acidobacteria representatives in different environments using molecular methods, it has been very difficult to isolate and culture them in laboratory, which limits the knowledge of their physiology and potential functions in the environment. To circumvent culture difficulties and identify potential traits that could suggest their ecological roles in soil, Ward et al. (2009) sequenced the complete genomes of three strains of the phylum Acidobacteria. The annotated characteristics included adaptation to low-nutrient conditions, capability for nitrate and nitrite reduction, presence of a large class of novel excreted proteins related to desiccation resistance, biofilm formation, and contribution to soil structure, as well as the production of novel antimicrobial compounds.

Although the soil bacterial community is stable at the phylum level, it is highly diverse at genera and species levels (Janssen 2006). *Conexibacter* was the most abundant genus identified in our samples. In 2010, the complete sequencing and annotation of the type species *C. woesei* was obtained by Pukall et al. (2010), who revealed a 6,359,369 bp long genome containing 5,950 protein-coding genes and 48 RNA genes. Briefly, members of *Conexibacter* are short rods*,* aerobic, motile with long flagella, Gram-positive, nonsporulating, and have a DNA G + C content of approximately 70%. Several distantly related uncultured bacterial clones have been detected in different environments, such as soil (Hartmann et al. 2009; Smith et al. 2006) and Fe-nodules of Quaternary sediments in Japan (Yoshida et al. 2008).

*Acidothermus* was the second most abundant genus in our samples. *A. cellulolyticus* is the type species and the only member of the genus and the family *Acidothermaceae.* It was first isolated from acidic hot springs at Yellowstone National Park (Mohagheghi et al. 1986) as an effort to improve the production of bioethanol from cellulosic biomass materials. The sequencing and detailed analysis of the genome of *A. cellulolyticus* (Barabote et al. 2008) revealed a new and diverse repertoire of enzymes involved in the degradation of plant biomass and highlighted the importance of this isolate for industrial applications. In summary, the *A. cellulolyticus* genome contains a set of genes encoding enzymes that degrade cellulose, xylans, and chitin, as well as glycogen and trehalose.

The main representative of Pseudomonadota in our samples was *Bradyrhizobium,* a bacterium of particular importance in agriculture because of its ability to fix nitrogen (Zhong et al. 2024). In addition to fixing nitrogen and transforming it into a form available to plants, which is a highly valuable feature for biofertilizers, they participate in the circulation of elements such as iron, phosphorus, sulfur, calcium and manganese, as well as in the decomposition of complex compounds present in the soil (Banasiewicz et al. 2025).

Another interesting genus of Pseudomonadota identified in our samples was *Roseiarcus*. The type species was isolated from a methanotrophic consortium obtained from acidic *Sphagnum* peat and represented a novel genus and species, for which the name *Roseiarcus fermentans* gen. nov., sp. nov. was proposed. The isolate also represents the first characterised member of a novel family within the class Alphapseudomonadota, Roseiarcaceae fam. nov. (Kulichevskaya et al. 2014).

The genus *Bryobacter* was the main representative of the phylum Acidobacteria in our samples. It is acid tolerant, strictly aerobic, slow growing, chemotrophic and has been isolated from acidic wetlands, soils, and quartzite caves in the Iron Quadrangle (Dedysh 2019; Lemes et al. 2021). *B. aggregatus* is the type species, which was the first taxonomically characterised member of subdivision 3 of Acidobacteria (Kulichevskaya et al. 2010).

In a study performed by Wang et al. (2022), the relative abundances of *Bryobacter* and *Candidatus* Solibacter increased in the rhizosphere soil of healthy sesame and were considered beneficial bacteria. The increased abundance of the strains was associated with a reduction in *Ralstonia*, the causative agent of bacterial wilt, in addition to other positive effects on the plant, such as those related to repair and cellular processes and continuous cropping signalling. *Candidatus* Solibacter is a heterotrophic, acidophilic bacterium that can reduce nitrate and nitrite but does not fix or denitrify nitrogen. It presents a wide range of enzymatic degradation machinery, which gives this bacterium the ability to degrade organic matter and utilize carbon sources (Du et al. 2017; Ward et al. 2009). The detailed mechanisms used by *Bryobacter* and *Candidatus* Solibacter to resist soil-borne diseases and increase plant resistance remain unknown. However, experiments conducted by Wang et al. (2022) revealed that healthy plant growth depends on an optimized bacterial soil community and a reasonable ratio of ammonium nitrate.

Many other bacteria whose biology and potential applications are yet to be discovered were identified in our study; however, the detailed description of so many genera is unfeasible. Our knowledge of Bacteria inhabiting *canga* and other extreme environments is scarce, and this is even more exacerbated regarding Archaea. Unlike initially thought, Archaea are currently recognized as a diverse group of microorganisms that are easily found in virtually any environment on the planet, where they coexist with members of Bacteria and Eukarya (Baker et al. 2020). Despite their ubiquity, we have just begun to understand these incredible microorganisms, mainly because of methodological limitations*.* Most of the currently known archaeal diversity is based on 16S rRNA gene amplicon sequencing. Nevertheless, Tahon et al. (2021), performing an *in-silico* analysis of the 16S rRNA gene primers currently available, reported that they failed to capture a significant fraction of several recently discovered archaeal clades. Therefore, the in-depth study of its diversity requires the design of specific primers based on meta (genomic) data.

### Statistical analyses of microbial community structure and physicochemical variables

Our analyses indicated that the differences in taxonomic composition and diversity between the samples were influenced by environmental factors. The microbial diversity (Shannon index) was best explained by a combination of physicochemical variables, such as OM, Mn, Fe, Al, pH and Mg, while the structure of the microbial community was influenced by the concentrations of Fe, Al and the CEC.

*Canga* is poorly developed and weakly structured, with a low nutrient content and relatively acidic pH between 4.8 and 5.1 (Schaefer et al. 2016). Elemental composition analyses have indicated that this environment is enriched in iron (57% of the total mass), aluminium (2–5%) and phosphorus (up to 0.8%) (Spier et al. 2003). Aluminium is typically present in *canga* and has been suggested to participate in the dissolution of primary oxides and in the process that leads to its formation (Levett et al. 2018).

The relationship between iron and bacteria in ferruginous duricrust is an important topic in the biogeochemistry and ecology of *canga*, especially in regions such as the Iron Quadrangle in Minas Gerais (Brazil). Iron cycling is important not only for its formation and maintenance but also for its ongoing evolution and recovery in post-mining rehabilitation attempts (Gagen et al. 2019). A wide variety of Fungi, Bacteria and Archaea can reduce iron oxides under various conditions, including anaerobic bacteria, fermentative and acidophilic organisms (Weber et al. 2006). Furthermore, in the presence of oxygen, ferrous iron rapidly oxidizes to ferric iron and precipitates as iron oxide minerals. The mutual action of iron-oxidizing and iron-reducing bacteria significantly enhances the cycling of iron and drives the continued dissolution and precipitation of iron in *canga* ecosystems (Levett et al. 2016; Parker et al. 2017).

### Functional prediction analyses

Functional prediction analyses performed on the most abundant genera identified in the samples revealed that the denitrification pathway is significantly increased, which is consistent with the high abundance of genera such as *Bradyrhizobium* and *Bryobacter*, both recognized for their roles in the nitrogen cycle. Denitrification is a crucial process for the loss of soil nitrogen (N) in gaseous forms, specifically nitric oxide (NO) and nitrous oxide (N_2_O). In addition to the loss of nutrients, the release of N_2_O into the atmosphere has ecological issues because it is a potent greenhouse gas. Nitrogen transformation and N_2_O production are complex processes that are conducted mainly by soil microbial chemical processes and are influenced by a variety of factors that are still misunderstood (Braker and Conrad 2011; Yin et al. 2023).

Denitrifying bacteria are usually favoured by low oxygenation, acidic pH, and soil organic carbon (SOC) availability (Xiao et al. 2021; Yang et al. 2018). The denitrification process is performed in four reaction steps, which include many enzymes that are regulated by different metalloenzymes (Henry et al. 2004). The relationship between iron oxidation and denitrification is of particular interest in this study. Chemodenitrification drives Fe(II) oxidation by nitrate-reducing bacteria under heterotrophic conditions, and secondary minerals formed after nitrate-reducing Fe(II) oxidation play a central role in carbon and nitrogen sequestration as well as heavy metal remediation (Pan et al. 2023). Several studies have reinforced the importance of further understanding the pathways of N_2_O production in highly acidic soils for improved agricultural practices and biotechnological applications.

### Ecological implications, acknowledges study limitations, and outlines priorities for future research

Extremophilic bacteria in ferruginous soils play key ecological roles by regulating metal biogeochemical cycles, mediating iron transformations, and influencing metal mobility and bioavailability. Despite harsh conditions, these environments can harbor unexpectedly diverse microbial communities, highlighting their importance as underexplored ecological hotspots and reservoirs of microbial diversity (Ripoll et al. 2025; Zurier et al. 2025). In this study, we identified several bacterial genera of scientific interest with potential biotechnological applications, such as *Acidothermus*, which is considered a promising source of novel enzymes for biomass degradation.

With respect to methodological limitations, it is important to acknowledge that many functional insights currently used are inferred from marker gene data rather than directly measured, which may limit the accuracy of ecological interpretations and depend on the representation of taxa in reference genome databases. Another important limitation is the lack of isolation of these microorganisms, which precludes controlled laboratory studies to validate their metabolic capabilities and ecological roles. Therefore, future research should prioritize the integration of multi-omics approaches (e.g., metagenomics, metatranscriptomics, and metabolomics) with experimental validation to better resolve the functional roles of these communities.

Understanding the microbial diversity inhabiting *canga* ecosystems, along with the physicochemical variables influencing their distribution, can contribute to a better understanding of these environments and support future strategies for their restoration and maintenance. In line with recent studies (Ripoll et al. 2025), our findings provide valuable insights into microbial ecology and emphasize the need for targeted conservation strategies for this biodiversity hotspot. Additionally, the sequencing data presented here, which are publicly available, may contribute to the development of sustainable biotechnological applications.

## Supplementary Information

Below is the link to the electronic supplementary material.

Supplementary file1 (PDF 236 kb)

Supplementary file2 (XLSX 17 kb)

Supplementary file3 (XLSX 1246 kb)

Supplementary file4 (XLSX 52 kb)

Supplementary file5 (PDF 232 kb)

Supplementary file6 (XLSX 16 kb)

Supplementary file7 (PDF 71 kb)

Supplementary file8 (PDF 202 kb)

## Data Availability

The raw sequencing data generated in this study are available in the NCBI Sequence Read Archive (SRA) under BioProject accession number PRJNA1303738 [https://www.ncbi.nlm.nih.gov/bioproject/PRJNA1303738]).
