# Supplementary material for: Bacterial diversity in ferruginous duricrust (canga) and the physicochemical variables affecting their prevalence, distribution and predicted metabolic pathways
Source: Antonie Van Leeuwenhoek. 2026 Apr 24;119(5):101. doi: 10.1007/s10482-026-02315-9 (PMC13109188; doi:10.1007/s10482-026-02315-9)

Bacterial diversity in ferruginous duricrust (canga) and the physicochemical variables affecting their prevalence, distribution and predicted metabolic pathways

Viviane Faria Morais Jotta[a] , Carla Alessandra Silva[a] , Glen Jasper Yupanqui García[b], Andrea Rodrigues Marques[c] , Andria dos Santos Freitas[d] , Aristóteles Góes-Neto[b] , Fernanda Badotti[a,e]*

a Postgraduate Program in Product and Process Technology, Centro Federal de Educação Tecnológica de Minas Gerais (CEFET-MG), Belo Horizonte, Minas Gerais, Brazil.

b Postgraduate Program in Bioinformatics, Universidade Federal de Minas Gerais (UFMG), Belo Horizonte, Minas Gerais, Brazil

c Department of Biological Sciences, Centro Federal de Educação Tecnológica de Minas Gerais (CEFET-MG), Belo Horizonte, Minas Gerais, Brazil

d Department of Genetic, Universidade Federal of Minas Gerais, Belo Horizonte, Minas Gerais, Brazil

e Department of Chemistry, Centro Federal de Educação Tecnológica de Minas Gerais (CEFET-MG), Belo Horizonte, Minas Gerais, Brazil

*Corresponding author: Av. Amazonas, 5.253, Nova Suiça, Belo Horizonte, MG, Brasil. CEP: 30.421-169, fbadotti@outlook.com

Online Resource 7 Boxplots of the soil physicochemical variables across the three transects (L1, L2, and L3)

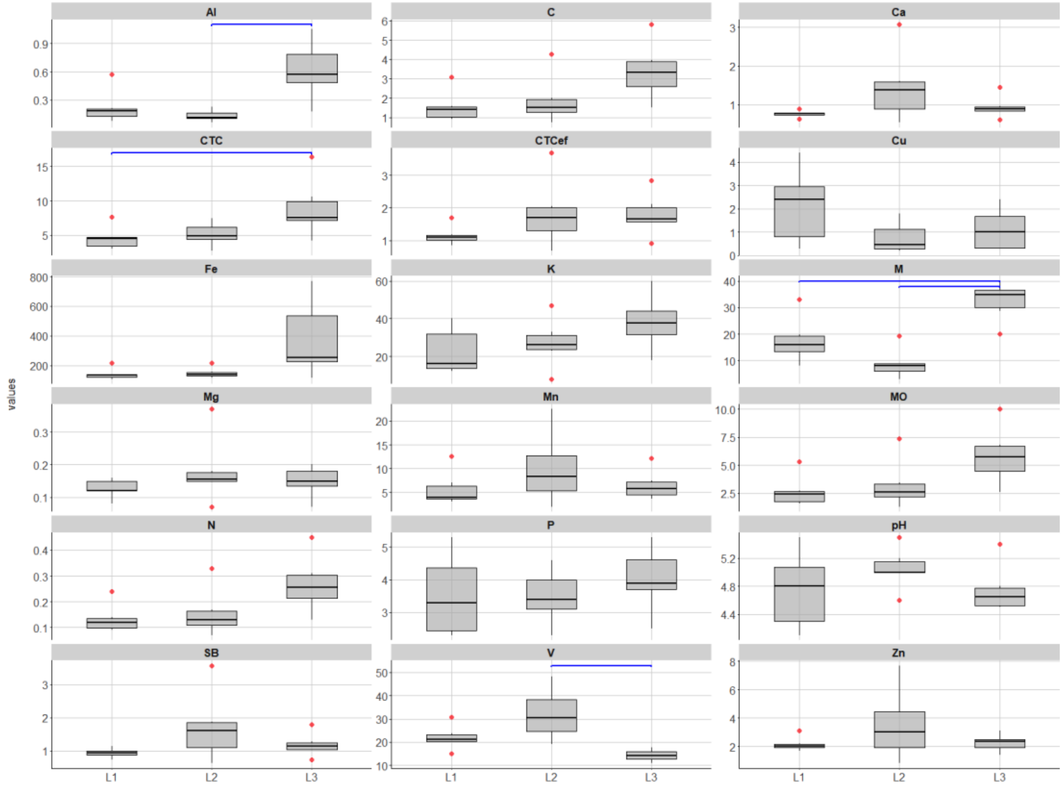

Supplement: Supplementary file 7 — Supplementary file7 (PDF 71 kb) [file 10482_2026_2315_MOESM7_ESM.pdf]
