# Supplementary material for: Bacterial diversity in ferruginous duricrust (canga) and the physicochemical variables affecting their prevalence, distribution and predicted metabolic pathways
Source: Antonie Van Leeuwenhoek. 2026 Apr 24;119(5):101. doi: 10.1007/s10482-026-02315-9 (PMC13109188; doi:10.1007/s10482-026-02315-9)
Supplement: Supplementary file 8 — Supplementary file8 (PDF 202 kb) [file 10482_2026_2315_MOESM8_ESM.pdf]

Bacterial diversity in ferruginous duricrust (canga) and the physicochemical variables affecting their prevalence, distribution and predicted metabolic pathways

Viviane Faria Morais Jotta[a] , Carla Alessandra Silva[a] , Glen Jasper Yupanqui García[b], Andrea Rodrigues Marques[c] , Andria dos Santos Freitas[d] , Aristóteles Góes-Neto[b] , Fernanda Badotti[a,e]*

a Postgraduate Program in Product and Process Technology, Centro Federal de Educação Tecnológica de Minas Gerais (CEFET-MG), Belo Horizonte, Minas Gerais, Brazil.
b Postgraduate Program in Bioinformatics, Universidade Federal de Minas Gerais (UFMG), Belo Horizonte, Minas Gerais, Brazil
c Department of Biological Sciences, Centro Federal de Educação Tecnológica de Minas Gerais (CEFET-MG), Belo Horizonte, Minas Gerais, Brazil
d Department of Genetic, Universidade Federal of Minas Gerais, Belo Horizonte, Minas Gerais, Brazil
e Department of Chemistry, Centro Federal de Educação Tecnológica de Minas Gerais (CEFET-MG), Belo Horizonte, Minas Gerais, Brazil

*Corresponding author: Av. Amazonas, 5.253, Nova Suiça, Belo Horizonte, MG, Brasil. CEP: 30.421-169, fbadotti@outlook.com

Online Resource 8 Functional prediction heatmap based on the total set of detected ASVs (see Materials and Methods for details). The prediction includes the phylogenetic positioning of the sequences, the reconstruction of ancestral states, and the estimation of the relative abundance of genes and metabolic pathways per sample.

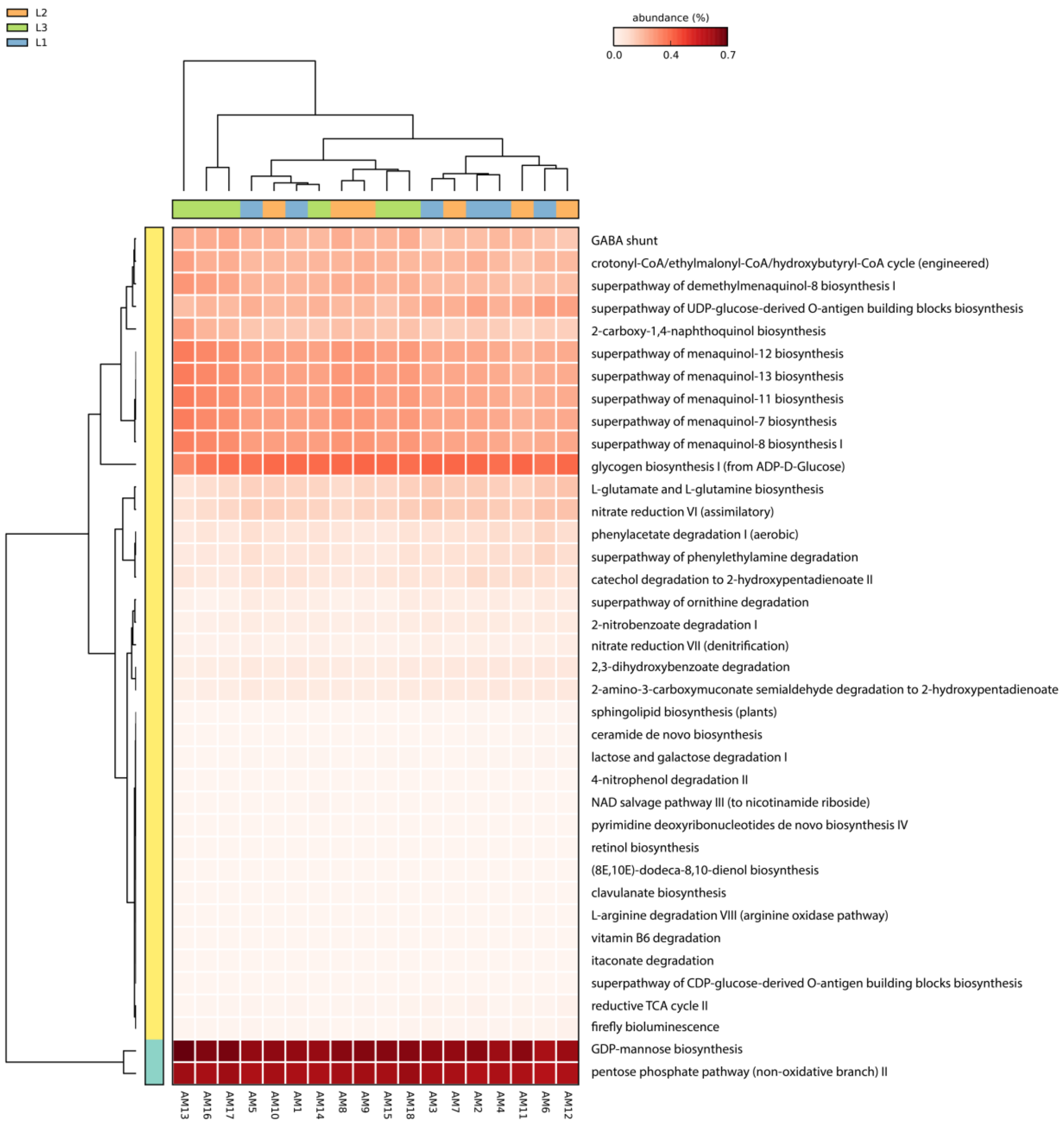
L2
L3
L1
abundance (%)
0.0
0.4
0.7
GABA shunt
crotonyl-CoA/ethylmalonyl-CoA/hydroxybutyryl-CoA cycle (engineered)
superpathway of demethylmenaquinol-8 biosynthesis I
superpathway of UDP-glucose-derived O-antigen building blocks biosynthesis
2-carboxy-1,4-naphthoquinol biosynthesis
superpathway of menaquinol-12 biosynthesis
superpathway of menaquinol-13 biosynthesis
superpathway of menaquinol-11 biosynthesis
superpathway of menaquinol-7 biosynthesis
superpathway of menaquinol-8 biosynthesis I
glycogen biosynthesis I (from ADP-D-Glucose)
L-glutamate and L-glutamine biosynthesis
nitrate reduction VI (assimilatory)
phenylacetate degradation I (aerobic)
superpathway of phenylethylamine degradation
catechol degradation to 2-hydroxypentadienoate II
superpathway of ornithine degradation
2-nitrobenzoate degradation I
nitrate reduction VII (denitrification)
2,3-dihydroxybenzoate degradation
2-amino-3-carboxymuconate semialdehyde degradation to 2-hydroxypentadienoate
sphingolipid biosynthesis (plants)
ceramide de novo biosynthesis
lactose and galactose degradation I
4-nitrophenol degradation II
NAD salvage pathway III (to nicotinamide riboside)
pyrimidine deoxyribonucleotides de novo biosynthesis IV
retinol biosynthesis
(8E,10E)-dodeca-8,10-dienol biosynthesis
clavulanate biosynthesis
L-arginine degradation VIII (arginine oxidase pathway)
vitamin B6 degradation
itaconate degradation
superpathway of CDP-glucose-derived O-antigen building blocks biosynthesis
reductive TCA cycle II
firefly bioluminescence
GDP-mannose biosynthesis
pentose phosphate pathway (non-oxidative branch) II
AM13
AM16
AM17
AM5
AM10
AM1
AM14
AM8
AM9
AM15
AM18
AM3
AM7
AM2
AM4
AM11
AM6
AM12
